# An improved estimation of tRNA expression to better elucidate the coevolution between tRNA abundance and codon usage in bacteria

**DOI:** 10.1038/s41598-019-39369-x

**Published:** 2019-02-28

**Authors:** Yulong Wei, Jordan R. Silke, Xuhua Xia

**Affiliations:** 1 Department of Biology, University of Ottawa, 30 Marie Curie, P.O. Box 450, Station A, Ottawa, Ontario Canada; 2 Ottawa Institute of Systems Biology, Ottawa, Ontario K1H 8M5 Canada

## Abstract

The degree to which codon usage can be explained by tRNA abundance in bacterial species is often inadequate, partly because differential tRNA abundance is often approximated by tRNA copy numbers. To better understand the coevolution between tRNA abundance and codon usage, we provide a better estimate of tRNA abundance by profiling tRNA mapped reads (tRNA tpm) using publicly available RNA Sequencing data. To emphasize the feasibility of our approach, we demonstrate that tRNA tpm is consistent with tRNA abundances derived from RNA fingerprinting experiments in *Escherichia coli*, *Bacillus subtilis*, and *Salmonella enterica*. Furthermore, we do not observe an appreciable reduction in tRNA sequencing efficiency due to post-transcriptional methylations in the seven bacteria studied. To determine optimal codons, we calculate codon usage in highly and lowly expressed genes determined by protein per transcript. We found that tRNA tpm is sensitive to identify more translationally optimal codons than gene copy number and early tRNA fingerprinting abundances. Additionally, tRNA tpm improves the predictive power of tRNA adaptation index over codon preference. Our results suggest that dependence of codon usage on tRNA availability is not always associated with species growth-rate. Conversely, tRNA availability is better optimized to codon usage in fast-growing than slow-growing species.

## Introduction

Codon optimization is critical to researchers seeking to improve protein production. Early experimental studies have shown that replacing rare codons with optimal ones increases protein yields in *Escherichia coli*^1,2^. The optimal codon within a given family is the most frequently used, especially in highly expressed genes (HEGs)^3–5^. In order to explain codon preference, early studies in *E*. *coli*^6–8^ have shown that codon usage coevolves with tRNA abundance. The availability of tRNAs influences the usage of corresponding codons; conversely, high usage of preferred codons drives up the availability of their decoding tRNAs^6,9^.

Additionally, tRNA-mediated codon usage bias has been broadly observed in a variety of organisms, including the gram-negative bacterium *Salmonella enterica* serovar typhimurium^10^, the gram-positive *Bacillus subtilis*^11^, eukaryotes such as yeast^10,12^, a variety of fungal and invertebrate mitochondrial genomes^13,14^, and viruses including HIV^15^, and bacteriophages^16,17^. Nonetheless, the nature of the relationship between tRNA abundance and codon usage across bacterial species has been the subject of debate among researchers, with some suggesting that tRNA availability is the main driving force of codon usage bias^18,19^ and others contending that the two are weakly correlated^20,21^.

A number of codon usage indices use tRNA gene copy as proxy of tRNA abundance to identify translationally optimal codons. These include the Codon Bias Index^22^, Frequency of Optimal Codons (F_op_)^10^, and tRNA Adaptation Index (tAI)^18^. All three of these indices define a translationally optimal codon as one that corresponds to the most abundant isoacceptor tRNA, with Codon Bias Index additionally incorporating gene expression information. Nevertheless, the use of tRNA gene copy is often undesirable. This is exemplified in *B*. *subtilis*^18^ in which tAI (based on tRNA gene copy) fails to accurately predict codon usage, although codon usage correlates strongly with early experimental tRNA abundance^11^ and conforms well to the selection-mutation-drift theory^21,23^. Moreover, while tRNA copy number is correlated with codon usage in a number of fast-growing bacterial species with a high variation in tRNA gene copy number, slow-growing ones exhibit little tRNA gene redundancy with many tRNA genes existing as a single copy. Resultantly, codon usage in such slow-growing species is poorly predicted by tRNA gene copy number^24^. For example, *Leptospira interrogans* and *Mycobacterium tuberculosis* have only 25 and 45 annotated tRNA genes according to the Genomic tRNA Database (GtRNAdb)^25^, respectively. Obviously, variation in codon usage cannot be well explained by tRNA gene redundancy if there is little variation in tRNA gene copy number. It stands to reason that the coevolutionary relationship between tRNA abundance and codon usage can be better characterized if tRNA abundance can be measured accurately.

To provide a better estimation for tRNA abundance in bacteria, we employed 14 publicly available RNA Sequencing (RNA-Seq) datasets, two for each of the seven species studied (*E*. *coli*, *S*. *enterica*, *B*. *subtilis*, *Bacteroides thetaiotaomicron*, *L*. *interrogans*, *M*. *tuberculosis*, and *Synechocystis* species). We quantified reads mapped to tRNA genes retrieved from GtRNAdb in transcripts per kilobase million (tpm) using kallisto^26^. To improve mapping efficacy, we have recently developed a new tool for processing RNA-Seq data, ARSDA^27^, that stores identical reads as single entries to drastically reduce data storage and computation time for analyzing large RNA-Seq datasets relative to previous methods^28,29^. These species were selected because their protein abundance data are available in PaxDb^30^, their growth rates are described on the basis of generation time (bacteria with >2.5 hour generation times are considered slow growing and all those with lower generation times are fast growing)^24^, and their RNA-Seq data are available (GEO Datasets).

A known issue with tRNA sequencing via standard Illumina protocols in eukaryotes^31,32^ is post-transcriptional methylation occurring at a number of specific tRNA sites. Two recent approaches (DM-tRNA-seq^32^ and ARM-seq^31^) of tRNA sequencing employ the *E*. *coli* derived AlkB demethylase enzyme to efficiently remove N^1^-methyladenosine (m^1^A), N^3^-methylcytosine (m^3^C) and N^1^-methylguanosine (m^1^G) structural modifications that hinder the activity of cDNA reverse transcriptase. Specifically, ARM-Seq^31^ demonstrates that wild-type AlkB alone is sufficient to remove all three of the aforementioned modifications and generate full length tRNA cDNA. These studies are consistent with a prior investigation that similarly concluded that AlkB could capably demethylate m^1^G^33^. It is important to note that both studies focus on eukaryotes, and AlkB treatments may not be necessary to remove tRNA methylations in bacteria that naturally encode their own AlkB homologs. Besides *E*. *coli*^31–34^, several lines of evidence suggest AlkB homologous proteins are present in other bacterial species^35–37^. Specifically, AlkB homologs are observed in *B*. *subtilis*^38^, *S*. *enterica*, *M*. *tuberculosis*^36,39^, *Synechocystis* sp.^36,40^, and species in *Leptospira*^36,39^ and *Bacteroides*^41^ genera. Nonetheless, bacterial tRNA sequencing efficiency is a point of investigation in this study.

To our knowledge, our results are the first to show that tRNA quantification by RNA-Seq data is well correlated with early tRNA abundance derived from RNA fingerprinting (hereafter referred as RNA fingerprinting abundance) reported previously in *E*. *coli*, *S*. *enterica* and *B*. *subtilis*^10–12,42^. Briefly, determining RNA fingerprinting abundances involve separating radiolabelled RNA by 2D gel electrophoresis followed by quantification of radioactivity. Despite the challenges associated with tRNA sequencing in yeast^31,43^ and mammals^31,32,44^, our results suggest that tRNA methylation may not appreciably influence tRNA sequencing efficiency in the bacterial species studied herein. We devised an integrated approach to show that tRNA tpm better predicts translationally optimal codons in *E*. *coli* than F_op_, and improves the predictive power of tAI over codon preference. We found that the dependence of codon preference on tRNA availability is not always stronger in fast-growing species, and optimal codons can be well explained by tRNA content in certain slow-growing species. Conversely, tRNA availability is better optimized to codon usage in highly expressed genes of fast-growing than slow-growing species.

## Results

### RNA-Seq mappings are consistent with tRNA abundance estimates

**Figure 1:**
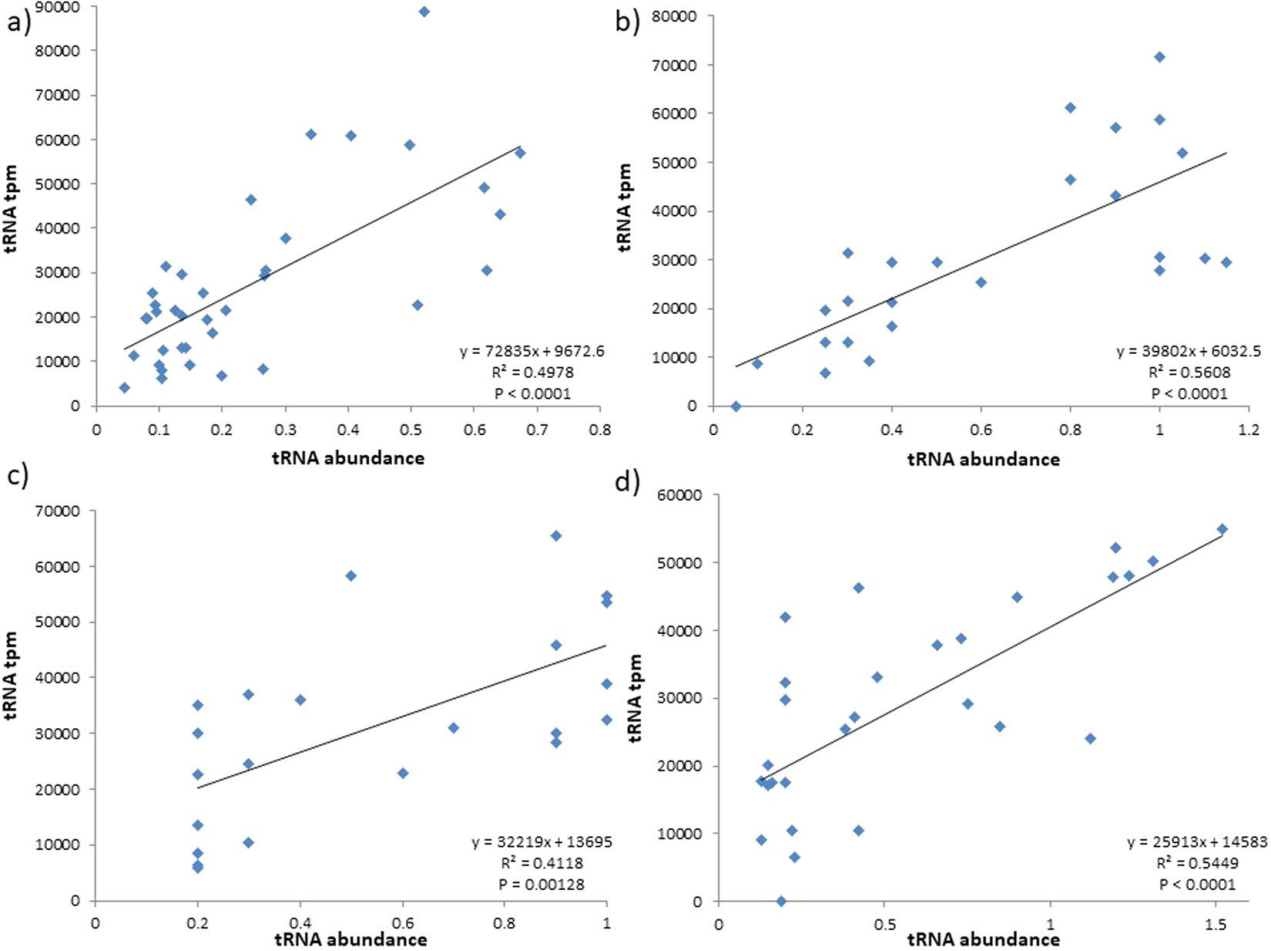
Comparison between tRNA tpm and fingerprinting abundance in *E*. *coli*, *S*. *enterica*, and *B*. *subtilis*. In panels (**a**) the averaged tRNA abundances across five growth phases retrieved from Dong, *et al*.^42^, (**b**,**c**) are tRNA abundances retrieved from Ikemura^10^, and in (**c**) the tRNA abundances retrieved from Kanaya, *et al*.^11^.

To accurately profile tRNA transcripts in tpm, we processed RNA-Seq data to remove adapters and low-quality sequences (See Materials and Methods for more detail) and quantified reads mapped to all unique tRNA sequences (Supplementary File S1) in tRNA tpm. To demonstrate the fidelity of tRNA tpm in bacteria, we compared these values with RNA fingerprinting abundances (Fig. 1, Supplementary File S2) previously reported in *E*. *coli*^10,42^, *S*. *enterica*^10^, and *B*. *subtilis*^11^. In all cases, tRNA tpm correlates with RNA fingerprinting abundance (Fig. 1: R^2^ > 0.4, P < 0.05).

### Documented tRNA methylation does not appreciably affect tRNA sequencing in bacteria studied

**Figure 2:**
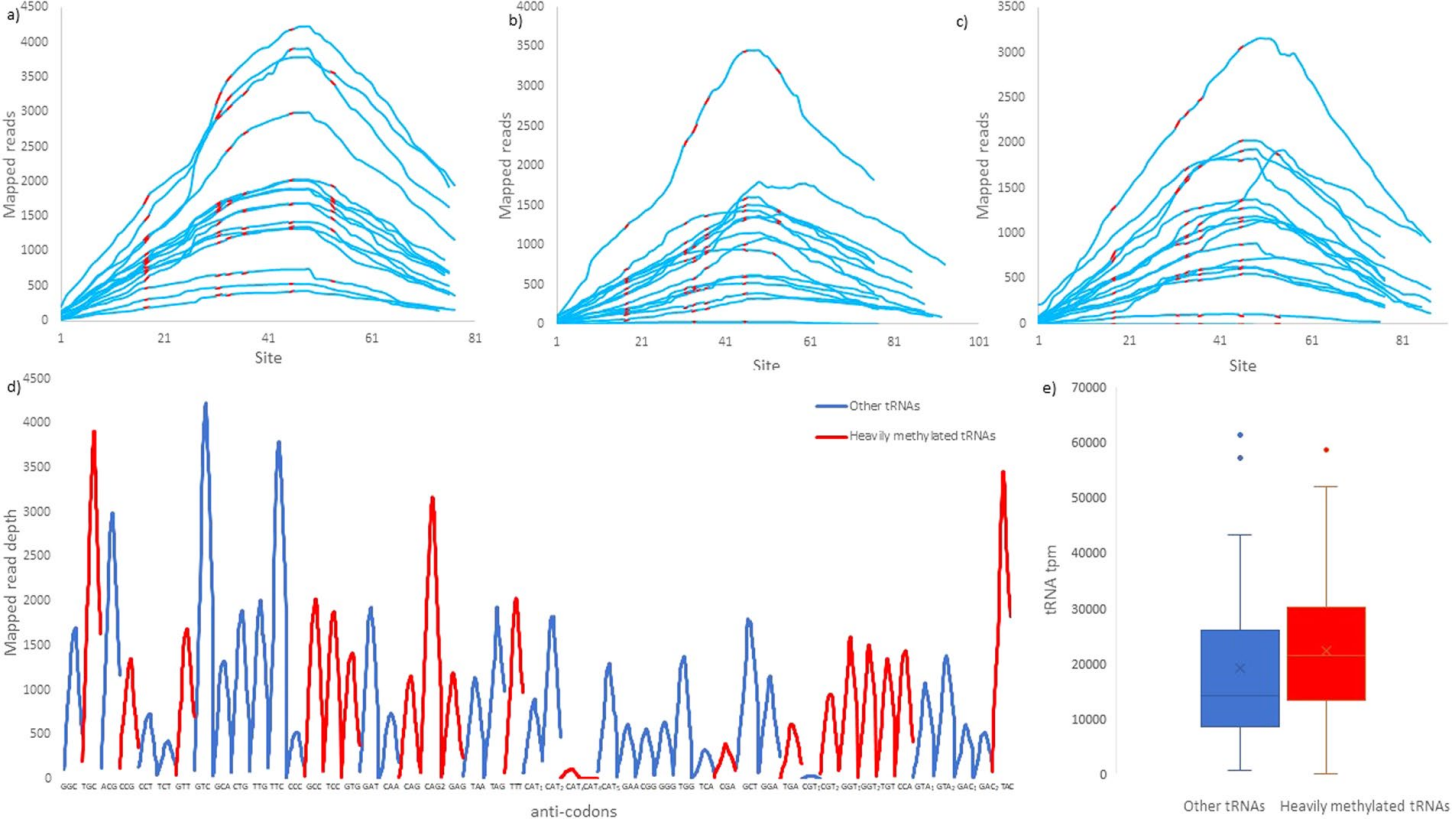
RNA-Seq read map for all 50 unique tRNA sequences in *E*. *coli*, split in three sets (**a**–**c**). Each line represents the read depth of entire sequence region of one unique tRNA sequence, with sites susceptible to methylation (m^2^G18, m^2^C32 or m^2^U32, m^5^U34 or m^2^C34 or cmo^5^U34, m^6^A37, m^7^G46 and m^5^U54) highlighted red. In (**d**) the distribution of mapped reads across the entire length of each unique tRNA sequence. Red indicates tRNAs that are potentially methylated at >4 sites (heavily methylated) and all others are highlighted blue. In (**e**) the distribution of total tRNA tpm in sets of heavily methylated and other tRNAs.

To investigate the potential effects of site-specific tRNA methylation on standard RNA-Seq experiments in bacteria, we visualized the RNA-Seq read depths for all seven species studied (Fig. 2, Supplementary File S1). In *E*. *coli*, read depths before and after documented tRNA methylation sites (18, 32, 34, 37, 46, and 54) in GenBank annotation (NC_000913, Supplementary File S1) do not vary substantially, and we observe no partial tRNA mappings (Fig. 2a–d) contrary to the “hard-stops” previously described in both yeast and human tRNAs in the absence of demethylation treatment^31^. Additionally, tpm values associated with the set of tRNAs that can be potentially modified at five or all six documented methylation sites do not differ substantially from the set of tRNAs that can be potentially modified at four or less sites (Fig. 2d,e; two-tailed Student’s t-test with unequal variance: P = 0.477). We define tRNAs that can potentially be methylated at >4 sites as heavily methylated with respect to other tRNAs. This cut-off was chosen because it divides the 50 unique tRNA sequences into two subsets of roughly equal size. Similarly, hard-stops were not observed at documented methylated sites in all six other bacteria studied (Supplementary File S1).

### An improved estimation of codon preference using tRNA tpm

To investigate how well tRNA tpm explains codon preference across bacterial lineages, we first designed an integrative approach to determine translationally optimal codons. We use two criteria to define what constitutes a translationally optimal codon: 1) it has the highest relative synonymous codon usage (RSCU)^45^ in HEGs, and 2) its RSCU in HEGs is greater than that in lowly expressed genes (LEGs). These two criteria combine the specifications of optimal codons defined by two previous studies: the first criteria is used by Novoa, *et al*.^19^ and the second is adapted from Rocha^24^ (HEGs vs. others). These criteria are also consistent with those used in the calculation of the Codon Adaptation Index^4^ and Index of Translation Elongation^5^. By our definition, there can be only one codon that is most translationally optimal within each synonymous group, consistent with the original definition of optimal codon determined by F_op_^10^. Thus, the characterized translationally optimal codons will increase elongation efficiency relative to others. The availability of protein abundance data in parts per million (ppm) for species studied herein and mRNA transcript abundance (in tpm) determined using kallisto^26^ enables us to calculate protein per transcript (ppm/tpm) in the identification of HEGs and LEGs (see Materials and Methods for more detail).

**Figure 3:**
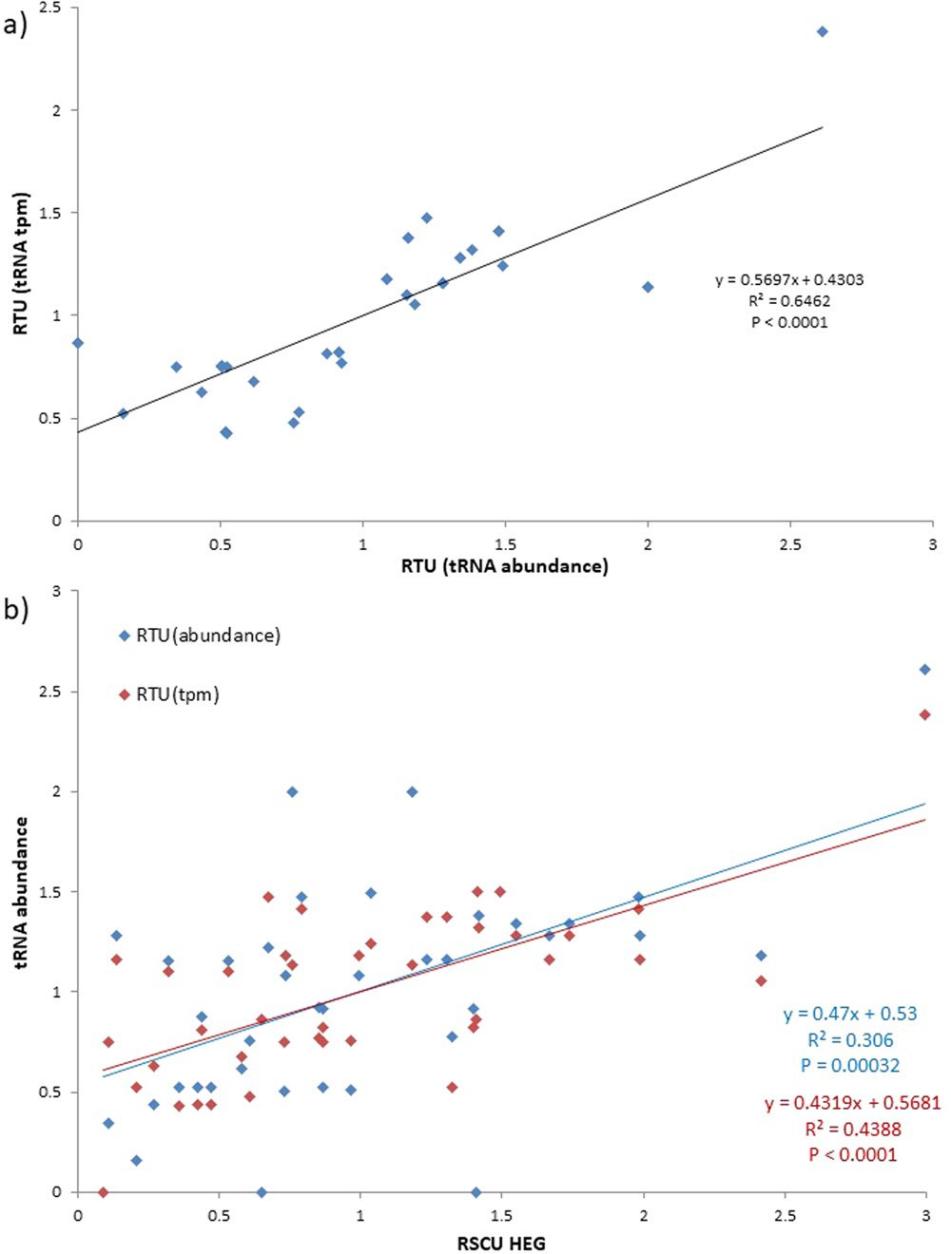
Relationship between (**a**) tRNA tpm and averaged tRNA abundance from RNA fingerprinting^42^ (tRNA abundance) in *E*. *coli*, and (**b**) RTUs and RSCU in *E*. *coli* HEGs.

To establish readable isoacceptor tRNA content, we consider all cognate and near-cognate interactions, as well as those enabled by anticodon modifications. Most studies^10,11,18,42^ consider anticodon-codon cognate (e.g., tRNA^Arg^_UGC_ reading GCA) and near-cognate (e.g., tRNA^Arg^_UGC_ reading GCG) pairings; while some^19,24^ also consider pairings allowed due to anticodon modifications (e.g., tRNA^Arg^_UmGC_ reading GCC and GCU) which increases the predictive influence of tRNA content on codon usage^19^. In order to explain RSCU, we adapt the idea of Relative tRNA Gene frequency from Novoa, *et al*.^19^ to derive Relative tRNA Usage (RTU) (see Materials and Methods for more detail). By considering tRNA abundance for synonymous codons, RTU improves tRNA tpm as an estimator of tRNA abundance (Fig. 3). In particular, the correlation between tRNA tpm and average tRNA abundance^42^ in *E*. *coli* (Fig. 1a; R^2^ = 0.498, P < 0.0001) improves if we consider their RTU values (Fig. 3a: R^2^ = 0.646, P < 0.0001). Furthermore, both RTU values correlate with codon usage (Fig. 3b).

Using RTU, we estimate how well translationally optimal codons match codons with the highest tRNA availability by adapting the four rules of codon-anticodon constraint^10^. Rule one states that codon usage is constrained by tRNA availability^46^. Rules two to four focus on specific base pairing efficiencies and they describe that cognate codon-anticodon pairs are generally more efficient and preferable relative to near-cognate wobble pairs^22,47–50^. Hence, we first rank synonymous codons by highest RTU, and then rank by cognate tRNA abundances (Supplementary File S2). Supplementary Fig. S1 provides a flowchart explaining the approach to predict translationally optimal codons by tRNA availability. For example, applying this two-step identification approach for Threonine codons in *E*. *coli*, we first select ACC and ACU since they both have the highest RTU and are both readable by the same tRNAs (tRNA^Thr^_GGU_ and tRNA^Thr^_UGU_) due to anticodon modification by ADATs^19^. Next, we rank codon preference by cognate tRNA abundance: tRNA tpm of the cognate tRNA^Thr^_GGU_ for ACC is 46537.7, and ACU is not decoded by a cognate tRNA. Thus, the predicted optimal codon based on tRNA availability and pairing constraints is ACC in Threonine. Indeed, ACC is the translationally optimal codon for Threonine based on our definition: it has the highest RSCU in HEGs (2.111), and (2) its RSCU HEG is higher than RSCU LEG (1.468). We extended this two-step approach to predict translationally optimal codons in all seven species (Table 1).

**Table 1:** Translationally optimal codons estimated for synonymous groups in seven bacterial species.

Amino acid^1^	Synonymous codons	*E*. *coli*	*S*. *enterica*	*B*. *subtilis*	*B*. *thetaiotaomicron*	*Synechocystis* sp.	*M*. *tuberculosis*	*L*. *interrogans*
Ala	GCA, GCC, GCG, GCU	—^3^	—	GCA^a^	GCU	**GCC**	GCC	GCA
Cys	UGC, UGU	**UGC** ^**b**^	**UGC**	**UGC**	UGU	—	**UGC**	—
Asp	GAC, GAU	—	—	—	GAU	—	**GAC**	—
Glu	GAA,GAG	GAA^ab^	**GAA**	**GAA** ^**a**^	GAA	**GAA**	**GAG**	**GAA**
Phe	UUC,UUU	**UUC** ^**ab**^	**UUC**	—	UUC	—	**UUC**	—
Gly	GGN	**GGC** ^**ab**^	**GGC**	**GGC**	GGU	GGU	**GGC** ^*^	—
His	CAC, CAU	**CAC** ^**b**^	—	—	—	—	**CAC**	—
Ile	AUA, AUC, AUU	**AUC** ^**ab**^	**AUC**	—	**AUC**	—	**AUC**	—
Lys	AAA, AAG	**AAA** ^**ab**^	**AAA**	**AAA** ^**a**^	AAA	—	**AAG**	—
Leu 2-fold^2^	UUA, UUG	**UUG** ^**b**^	**UUG**	UUA^a^	—	UUG	**UUG**	—
Leu 4-fold	CUA, CUC, CUG, CUU	**CUG** ^**a**^	**CUG**	CUU	—	CUG^*^	**CUG**	—
Asn	AAC, AAU	**AAC** ^**ab**^	**AAC**	—	—	—	**AAC**	—
Pro	CCA, CCC, CCG, CCU	**CCG** ^*ab^	**CCG**	—	—	CCC^*^	**CCG**	CCU
Gln	CAA, CAG	**CAG** ^**ab**^	**CAG**	**CAA** ^**a**^	—	—	**CAG**	—
Arg 2-fold	AGA, AGG	AGA^a^	AGA	AGA	AGA	**AGG**	**AGG**	**AGA**
Arg 4-fold	CGA, CGC, CGG, CGU	**CGU** ^**a**^	**CGU**	CGC	**CGU**	**CGG** ^*^	CGC	CGU
Serine 2-fold	AGC, AGU	**AGC** ^**b**^	**AGC**	**AGC**	—	—	**AGC**	—
Serine 4-fold	UCA, UCC, UCG, UCU	UCU	**UCC**	UCA	UCU	UCC	—	UCU
Thr	ACA, ACC, ACG, ACU	**ACC** ^**ab**^	**ACC**	ACA^a^	ACU	**ACC**	**ACC**	ACU
Val	GUA, GUC, GUG, GUU	—		GUU	**GUA**	—	—	
Tyr	UAC, UAU	—		—	—	—	**UAC**	

Bold are translationally optimal codons that also have the highest tRNA availability estimated by tpm from two independent RNA-Seq datasets.

^1^Two amino acids (Met and Trp) are omitted because they are each encoded by a single codon.

^2^The 6-fold degenerate codon families (Leu, Arg, and Ser) are broken into 2 and 4-fold families because of differences in the first codon base.

^3^An optimal codon cannot be determined by our definition (e.g., RSCU HEG < RSCU LEG violates the second criterion).

^*^Predicted preferred codons match optimal codons in only one of the two RNA-Seq data analyzed.

^a^Translationally optimal codons match optimal codons determined by F_op_ in Ikemura^10^ and Kanaya, *et al*.^11^.

^b^Translationally optimal codons identified using average tRNA abundance from RNA fingerprinting approach, retrieved from Dong, *et al*.^42^.

Many studies have demonstrated that bacterial tRNA abundance fluctuates due to growth phase and culture conditions such as temperature and media^42,51–53^. Hence, for each species, we have acquired tRNA tpm values from two independent, but experimentally consistent (i.e., all strains are wildtype, and all cultures are taken during log-phase growth) RNA-Seq datasets (Table 2) that were prepared for sequencing on the same platform (Illumina) to verify consistency in predicting translationally optimal codons using tRNA tpm (Table 1).

**Table 2:** The seven bacterial species studied herein due to their availability of protein abundance, growth rate and RNA-Seq data.

Species	Strain	Growth Rate^a^	NCBI Accession	Experiment ID^*^
*Bacteroides thetaiotaomicron*	VPI-5482	Slow	NC_004663	SRX020805, SRX860738
*Bacillus subtilis*	168	Fast	NC_000964	SRX515181, SRX2804667
*Escherichia coli*	K-12	Fast	NC_000913	SRX515174, SRX669653
*Leptospira interrogans*	Fiocruz L1-130	Slow	AE016823	SRX2448246, SRX405952
*Mycobacterium tuberculosis*	H37Rv	Slow	NC_000962	SRX1372108, SRX4374910
*Salmonella enterica*	LT2	Fast	NC_003197	SRX1638989, SRX1258668
*Synechocystis* sp.	PCC 6803	Slow	NC_017277	SRX347145, SRX4145044

^a^Information on species growth-rate are retrieved from Rocha^24^.

^*^All selected datasets have matching species strains between protein abundance, GtRNAdb, NCBI and RNA-Seq data, with the exception of *L*. *interrogans* (SRX2448246) due to the lack of a second RNA-Seq data in GEO Datasets for strain Fiocruz L1-130. All cultures in RNA-Seq experiments were isolated during log phase of growth. The first listed SRX dataset was selected for all analyses, and both were used for Table 1 and Fig. S4.

### Implementing tRNA tpm in tAI calculation improves the non-parametric *S* correlation

We calculated tAI using gene copy number and tRNA tpm (See Materials and Methods for more detail, Supplementary Fig. S2), and Table 3 shows the non-parametric regression *S* which reflects the correlation between tAI values and effective number of codons^54^ (corrected for silent substitutions^18^). For six out of seven species studied, *S’* calculated with tRNA tpm is higher than *S* calculated using tRNA gene copy number (Table 3). Hence, tAI’ values calculated using tRNA tpm better correlate with codon usage than tAI values obtained from tRNA gene copy number (two-tailed Student’s t-test with unequal variance: P < 0.0001; Supplementary Fig. S2) in all species except *L*. *interrogans*. Note that *S* values calculated herein use non-hypothetical and non-pseudo genes, whereas those originally calculated (*S*_0_)^18^ use all coding DNA sequences, although value ranks stay consistent (Table 3).

**Table 3:** Non-parametric regression *S* correlations between tAI values and effective number of codons.

Species	*S*_*0*_	*S*	*S'*
*E*. *coli*	0.70^a^	0.61^b^	0.71^c^
*S*. *enterica*	0.63	0.59	0.69
*B*. *thetaiotaomicron*	0.55	0.4	0.62
*Synechocystis sp*.	0.38	0.27	0.5
*B*. *subtilis*	−0.01	0.18	0.33
*M*. *tuberculosis*	−0.04	0.1	0.13
*L*. *interrogans*	N/A	0.23	0.2

^a^S values retrieved from dos Reis, *et al*.^18^, calculated using all coding DNA sequences.

^b^S values calculated using tRNA gene copy number, using genes having non-zero protein abundances.

^c^S values calculated using tRNA tpm, using genes having non-zero protein abundances.

### Abundance of tRNA depends on codon usage in fast-growing species

Codon usage preferences can also drive up tRNA content^6,24^. For each tRNA species (distinguished by anticodon), we define its readable codon usage as the sum usage of its cognate and near-cognate codons (e.g., the readable codon usage of tRNA^Ala^_GGC_ = GCC usage + GCU usage). Readable codon usage in HEGs better explains tRNA tpm in fast-growing species than in slow-growing species. More specifically, readable codon usage better correlates with tRNA tpm in *E*. *coli*, *S*. *enterica*, and *B*. *subtilis*, than in *B*. *thetaiotaomicron*, *Synechocystis* sp., *M*. *tuberculosis*, and *L*. *interrogans* (Supplementary Fig. S2).

## Discussion

Two approaches have been taken to characterize the effect of tRNA on codon usage. The first tests whether gain or loss of tRNA genes will lead to predicted changes in codon usage^55–57^. In tunicates and bivalves, an additional tRNA^Met/UAU^ gene is present in the mitochondrial genome. One would expect that the additional tRNA^Met/UAU^ would favor increased usage of AUA codons, and this expectation is empirically substantiated^55,56^. One may also reason that, if a bacteriophage encodes many tRNA genes in its own genome, especially when these tRNAs are rare in the host, then the phage codon usage will be less dependent on the host tRNA pool. This expectation is also consistent with empirical evidence^16,58^. The second approach quantifies within-species association of codon usage with tRNA abundance which is often approximated by tRNA gene copy number, but this proxy has two key shortcomings. First, we do not know if it is generally true that tRNA gene copy number serves as a good proxy for tRNA abundance. Second, some bacterial species, such as *M*. *tuberculosis*, have only a single tRNA gene for all anticodons. In such cases, we cannot use tRNA gene copy number as a proxy of abundance because of its lack of variability. Thus, accurate quantification of tRNA abundance is crucial for detecting codon adaptation.

Our RNA-Seq-based analysis shows that snapshots of bacterial tRNA pools can be captured using RNA-Seq profiling in spite of claims that standard Illumina RNA Sequencing protocols inefficiently quantify tRNAs in eukaryotes^32,43,44^ due to a number of modifications that increase the stability of tRNA secondary structure. While some tRNA modifications are shared among the three kingdoms of life, others are kingdom specific^59^. Nonetheless, tRNA post-transcriptional methylation is extensive in Eubacteria^59^, and we acknowledge that tRNA tpm values may underestimate tRNA abundances since tRNA is highly structured and difficult to denature. Despite this, we do not observe any drastic read count drops or hard-stops in mapped reads at or flanking documented methylated sites in the seven species studied here, nor do we see partially mapped tRNA regions (Fig. 2a–c; Supplementary File S1) that were commonly observed in untreated tRNA sequencing data in Eukaryotes^31^. A caveat is the presence of a hard-stop in most tRNAs at site 50 for *B*. *subtilis* (SRX2804667), *Synechocystis* sp. (SRX4145044) and *L*. *interrogans* (SRX2448246) (Supplementary File S1). However, there is no documented methyltransferase activity acting at this site for these species, and the drop in mapped reads is only observed in one of the two SRX datasets retrieved for each species. Nonetheless, we acknowledge that tRNA sequencing may be potentially enhanced with demethylase treatments^31,32^ that should be employed in future tRNA-Seq studies in bacteria.

Because eukaryotic tRNA read mapping abundances are considerably higher in demethylated samples than untreated samples^31^, we expected that our read mapping abundances may be similarly impacted by tRNA methylation. In light of this, we considered all annotated methylation sites in bacterial tRNAs, even though they may not be methylated at all times due to structural constraints. Surprisingly, our observations reveal that read mapping abundances of *E*. *coli* tRNAs that are potentially heavily methylated do not differ from those that are susceptible to methylation at fewer than five methylation sites (Fig. 2e). This suggests that the requirement for demethylase treatment prior to tRNA sequencing in bacterial species with functional AlkB demethylase homologs may be more relaxed, especially since demethylation treatments in current eukaryotic tRNA sequencing approaches are bacterial AlkB-facilitated^31,32^.

In all bacteria, we combined our RNA quantification approach with available protein abundances to determine the most translationally optimal codon in 21 codon groups (Table 1) based on codon usage differences between HEG and LEG subsets. These subsets of genes are established based on protein per transcript (Supplementary Table S1, File S3), with the aim of providing a more accurate estimate of translation efficiency from protein abundance by decoupling rates of transcription. However, species-specific translationally optimal codons cannot always be established because the two described criteria are violated in some groups (e.g., the codon with the highest RSCU is more over-represented in LEGs than HEGs). In these cases, there is no evidence that the most abundantly used synonymous codon would contribute to increase translation efficiency. In particular, *L*. *interrogans* represents a slow-growing species wherein codon optimization is very poor (only seven translationally optimal codons can be characterized in 21 codon groups).

Our tRNA quantification approach better predicts translationally optimal codons over F_op_^10^. In *E*. *coli*, synonymous codons with the highest tRNA tpm (ranked by highest TPU followed by highest cognate tRNA abundance) match 15 out of 17 translationally optimal codons, but 13 out of 17 when we replace tRNA tpm with averaged RNA fingerprinting abundance retrieved from Dong, *et al*.^42^ (Table 1). In contrast, F_op_ determines 12 such translationally optimal codons^10^ of which AGA (Arg) is the only codon that our method does not predict to be optimal (Table 1). Additionally, all translationally optimal codons determined herein are consistent with optimal codons determined by F_op_, except in the Serine 4-fold family where F_op_ predicts UCC whereas we predict UCU to be optimal. In the case of *B*. *subtilis*, both tRNA tpm and F_op_ determine six translationally optimal codons (Table 1). It is worth mentioning that F_op_ determines 16 optimal codons in *B*. *subtilis*^11^, but most may not be translationally optimal. For example, CCA (Pro) was determined to be an optimal codon, but CCG (Pro) is substantially more preferred than CCA (Pro) (RSCU in HEGs are 1.735 and 0.782, respectively).

Nonetheless, bacterial tRNA abundance may not fully explain the variation in usage of all 61 sense codons (Supplementary Fig. S4). First, codon preference cannot always be inferred reliably from tRNA gene redundancy or experimentally measured tRNA abundance. For example, inosine is expected to pair best with C and U, but less with A (presumably because of the bulky I/A pairing involving two purines)^60^. Second, what matters in translation elongation is the availability of charged tRNAs. It is difficult to determine the level of charged tRNAs, and researchers typically would use transcriptionally determined tRNAs or even the number of tRNA genes in the genome as a proxy of charged tRNAs. Unfortunately, the abundance of tRNAs does not always reflect the abundance of charged tRNA^61^. Lastly, other factors such as mutation bias^21,62–65^ may exert more pressure on codon usage in certain species.

Conversely, the variation in tRNA tpm is better explained by codon usage (Supplementary Fig. S3) in fast-growing (*E*. *coli*, *B*. *subtilis* and *S*. *enterica*) than in slow-growing species (*B*. *thetaiotaomicron*, *L*. *interrogans*, *M*. *tuberculosis* and *Synechocystis* sp.). This result supports the theory that tRNA translation machinery is better optimized to codon usage in fast-growing than slow-growing species^9,24^. Indeed, duplicating tRNA genes is an effective way to elevate transcript abundance in species that grow and replicate rapidly^10–12,42^, but not in slow-growing species (Supplementary Fig. S5).

One potentially important implementation of tRNA tpm is in the calculation of tAI. Our results (Table 3, Supplementary Fig. S2) show that tAI’ (calculated using tRNA tpm) better explains effective number of codons than tAI (calculated using tRNA gene copy number) for all species studied except *L*. *interrogans*. Considering *S* and *S*_0_ calculated using tRNA gene copy numbers, their differences are likely due to our usage of the subset of non-hypothetical and non-pseudo genes that have protein abundance values^30^ (*S*) whereas all DNA coding sequences (including hypothetical and pseudo genes) were used in the original calculation^18^ (*S*_0_). Additionally, both the GtRNAdb^25,66^ and DNA coding sequences (GenBank annotations) have been continuously curated since 2004. Lastly, only wobble base pairings were considered in the original introduction of tAI^18,67^; whereas we have also considered possible anticodon modifications^19,24^ that further relax codon pairing. These differences improve the calculation of the *S* correlation using tRNA copy numbers, notably in *B*. *subtilis* and *M*. *tuberculosis*. In contrast, the originally calculated negative *S*_0_ correlation for *B*. *subtilis* was a major shortcoming of the tAI method^18^ and was criticized^21^ for suggesting a lack of selective pressure exerted by tRNA abundance on codon preference in this species.

In the case of *M*. *tuberculosis*, our tRNA quantification approach is much more sensitive to determining tRNA-mediated codon bias than tAI. The *S* correlations are consistently the lowest for *M*. *tuberculosis* (Table 3), yet we identified 17 out of 19 translationally optimal codons using tRNA tpm (Table 1). Our method recaptures the “weak but significant codon usage preference” previous reported^21,68^ in this slow-growing species, and show that the degree to which tRNA availability explains optimal codon usage is species-specific and does not always depend on growth-rate.

We studied the coevolution between codon usage and tRNA abundance in three fast-growing species (*E*. *coli*, *S*. *enterica*, and *B*. *subtilis*) and four slow-growing species (*B*. *thetaiotaomicron*, *L*. *interrogans*, *M*. *tuberculosis*, and *Synechocystis* sp.). Our findings indicate that tRNA quantification by tpm offers better predictions of translationally optimal codons over F_op_ in *E*. *coli*, and improves the calculation of tAI to better reflect codon preference in all species studied except *L*. *interrogans*. The usage of translationally optimal codons can be well explained by relative tRNA tpm in *E*. *coli* and *S*. *enterica*; however, both tRNA tpm and RNA fingerprinting abundances^11^ offer weaker explanations for codon preference in *B*. *subtilis*. The influence of tRNA availability on codon bias is not always stronger in fast-growing species, and optimal codons can be well explained by tRNA content in certain slow-growing species such as *M*. *tuberculosis*. Conversely, the tRNA translation machinery is better optimized to codon usage in HEGs of fast-growing than slow-growing species.

## Materials and Methods

### Processing genomic, proteomic and RNA-seq data

We retrieved the annotated genomes (Table 2) for three fast growing species (*E*. *coli*, *B*. *subtilis*, and *S*. *enterica*) and four slow-growing species (*B*. *thetaiotaomicron*, *L*. *interrogans*, *M*. *tuberculosis*, and *Synechocystis* sp.) in GenBank format from the National Center for Biotechnology Information (NCBI) database (http://www.ncbi.nlm.nih.gov). For each species, all documented tRNA methyltransferase genes were retrieved from GenBank annotations (Supplementary File S1).

Protein abundance data corresponding with each of these species were retrieved from PaxDb 4.0^30^ https://pax-db.org/ and abundance values were associated with GeneIDs retrieved from the genomes using DAMBE 7^69^. The integrated protein abundance dataset was taken when available.

RNA-Seq runs of wildtype species were fetched from GEO DataSets (https://www.ncbi.nlm.nih.gov/gds/) in FASTQ format. FASTQ files were converted to FASTQ+ format using ARSDA 1.1^27^ in order to reduce file sizes by grouping identical reads under a single ID while retaining the copy number for each read (in the format S<read#>_<copy#>). The FASTQ+ data was then processed using both CutAdapt 1.17^70^ and Trimmomatic 0.38^71^ to remove flanking adapter sequences and purge low quality reads. For experiments that use the oligo(dT)-adapter primer for cDNA synthesis, RNA fragments are first poly-adenylated at the 3′ end. In these cases, we set CutAdapt to recognize “AAAAA”. We also used CutAdapt to recognize and remove all possible adapters in experiments that used custom adapters, with 10% mismatch error rate. When the adapters conformed to standard Illumina protocols, we simply used the “ILLUMINACLIP” function built into Trimmomatic to trim the relevant library of adapters from reads (ILLUMINACLIP: <adapters.fa> :2:20:6). After adapters were trimmed, we retained reads that were a minimum of 25 nt long (-m 25 in CutAdapt or MINLEN:25 in Trimmomatic) to mitigate bias in expression levels^72^. We filtered trimmed reads to remove poor quality sequences with average Phred scores lower than 30 (0.1% probability of a base calling error)^73^.

### RNA-Seq read mapping for tRNAs and mRNAs

We retrieved the sequences of all genomically encoded tRNAs for each organism from the Genomic tRNA Database (GtRNAdb 2.0^66^) in FASTA format and removed predicted pseudo-tRNAs and those with unspecified anticodons. The FASTA files containing tRNA sequences were read into DAMBE to represent identical sequences with one ID indicating the number of identical copies. Since mature tRNAs are modified to have 5′-CCA-3′ appended to their 3′ end, we manually added CCA to sequences lacking this motif^32^. The modified tRNA FASTA files for all species were indexed and the associated RNA-Seq reads from processed FASTQ+ files were pseudo-aligned to each tRNA index and tRNA tpm was quantified using kallisto v0.44.0^26^. The tRNA pseudo-alignments for each species were subsequently sorted and site-specific depth values were generated for each tRNA using the sorted pseudo-alignments via the ‘sort’ and ‘depth’ commands from SAMtools^74^, respectively.

Similarly, all non-pseudo and non-hypothetical DNA coding sequences with non-zero protein abundance values were retrieved using DAMBE in FASTA format and indexed. The associated RNA-Seq reads from processed FASTQ+ files were pseudo-aligned to each mRNA index and mRNA tpm values quantified using kallisto.

### Determination of translationally highly and lowly expressed genes by protein per transcript

Protein per transcript (ppm/tpm) was estimated by taking gene protein abundance (ppm) divided by its mRNA tpm, for both RNA-Seq datasets in each species except *B*. *subtilis*. For *B*. *subtilis*, protein per transcript was obtained with only SRX515181 dataset, because SRX2804667 MiSeq experimental protocol effectively removes large transcripts to study tRNAs^75^. From each dataset, genes with top and bottom 30% ppm/tpm values were selected (Supplementary File S3). A gene is considered to be highly expressed if the gene ID is found in both gene sets for each species; the same was done to identify lowly expressed genes from the bottom 30% ppm/tpm gene sets (Supplementary File S3, Table S1). To verify the validity of this approach, we determined the number of ribosomal protein (30S and 50S subunit) genes that are present in each gene sets. We observed a great number of ribosomal protein genes in the top 30% ppm/tpm gene sets and nearly none in the bottom 30% ppm/tpm gene sets (Supplementary Table S1). This is expected because ribosomal protein genes are commonly accepted and used as highly expressed genes^4,24^.

### Computation of relative synonymous codon usage and tRNA usage metrics

Relative synonymous codon usage (RSCU)^45^ values were computed for each species by loading HEGs and LEGs (Supplementary File S3) into DAMBE and selecting “Seq. Analysis” > “Codon Usage” > “Relative synonymous codon usage”. DAMBE’s implementation of the RSCU computation automatically splits 6-fold degenerate codon families into a 2-fold and 4-fold degenerate family based on difference at the first codon position.

To acquire relative tRNA usage for each synonymous codon (RTU), we adapt the RSCU formula (1) in the same way that Relative tRNA Gene frequency was employed by Novoa, *et al*.^19^ using tRNA gene copy number:

1 $$RT{U}_{i}=\frac{tpm\,of\,tRN{A}_{i}}{\frac{{\sum }_{i}^{n}tRN{A}_{i}}{n}}$$

where *i* is any codon within a 2 or 4-fold degenerate codon family, and *n* is the total number of codons in the synonymous group. RSCU and RTU are both calculated by breaking 6-fold codon families (Arginine, Leucine, and Serine) into 2 and 4-fold groups (e.g., 4-fold CGN (arg) and 2-fold AGN (arg)). Methionine and Tryptophan have been omitted from RSCU and RTU calculation because they are encoded by a single codon (RSCU and RTU = 1). Similarly, codon groups with RTU = 1 have been omitted from plots when applicable (Supplementary Fig. S4), because RTU will not estimate RSCU for these codons. All correlation coefficients (R^2^) are calculated by taking the square of Pearson’s correlation r (Figs 1 and 3, Supplementary Figs S3–5).

### Computation of tAI and correlation *S*

We first calculated tAI using the original formulation of the model^18,67^ which considers the copy number of all isoacceptor tRNAs for each codon via the author’s tAI R package version 0.2 (https://github.com/mariodosreis/tai) for all species in this study. We additionally computed a modified version of tAI (tAI’) which uses the summed tpm values associated with codon-specific isoacceptor tRNAs in lieu of tRNA copy number. Rather than using all annotated DNA coding sequences in these calculations, as was done originally^18^, we only considered genes with non-zero protein abundance values from the integrated datasets stored in PaxDb. The tAI and tAI′ values for each species were plotted (Supplementary Fig. S2) against the effective number of codons corrected for silent substitutions at the third codon position (f[GC3s] - Nc) to determine the *S* and *S’* correlation coefficients, respectively.

## Supplementary information

Supplementary File S1

Supplementary File S2

Supplementary File S3

Supplementary File S4

## Data Availability

Supplementary File S1 contains RNA-Seq read depths and tRNA methylation profile. Supplementary File S2 contains identifications of translationally optimal codons and tRNA abundances (gene copy, tpm and fingerprinting data) and tRNA quantification approaches. Supplementary File S3 contains protein per transcript data, protein abundance information, identified translationally HEGs and LEGs. Supplementary File S4 contains Supplementary Figs S1–S5 and Table S1.
