# Supplementary material for: An improved estimation of tRNA expression to better elucidate the coevolution between tRNA abundance and codon usage in bacteria
Source: Sci Rep. 2019 Feb 28;9:3184. doi: 10.1038/s41598-019-39369-x (PMC6395768; doi:10.1038/s41598-019-39369-x)
Supplement: Supplementary file 4 — Supplementary File S4 [file 41598_2019_39369_MOESM4_ESM.pdf]

An improved estimation of tRNA expression to better elucidate the coevolution between tRNA abundance and codon usage in bacteria

Yulong Wei[1,+], Jordan R. Silke[1,+], Xuhua Xia[1,2,*]

1. Department of Biology, University of Ottawa, 30 Marie Curie, P.O. Box 450, Station A, Ottawa, Ontario, Canada, K1N 6N5. Tel: (613) 562-5800 ext. 6886, Fax: (613) 562-5486.

2. Ottawa Institute of Systems Biology, Ottawa, Ontario, Canada K1H 8M5.

* Corresponding author E-mail: xxia@uottawa.ca
+ Equal contribution.

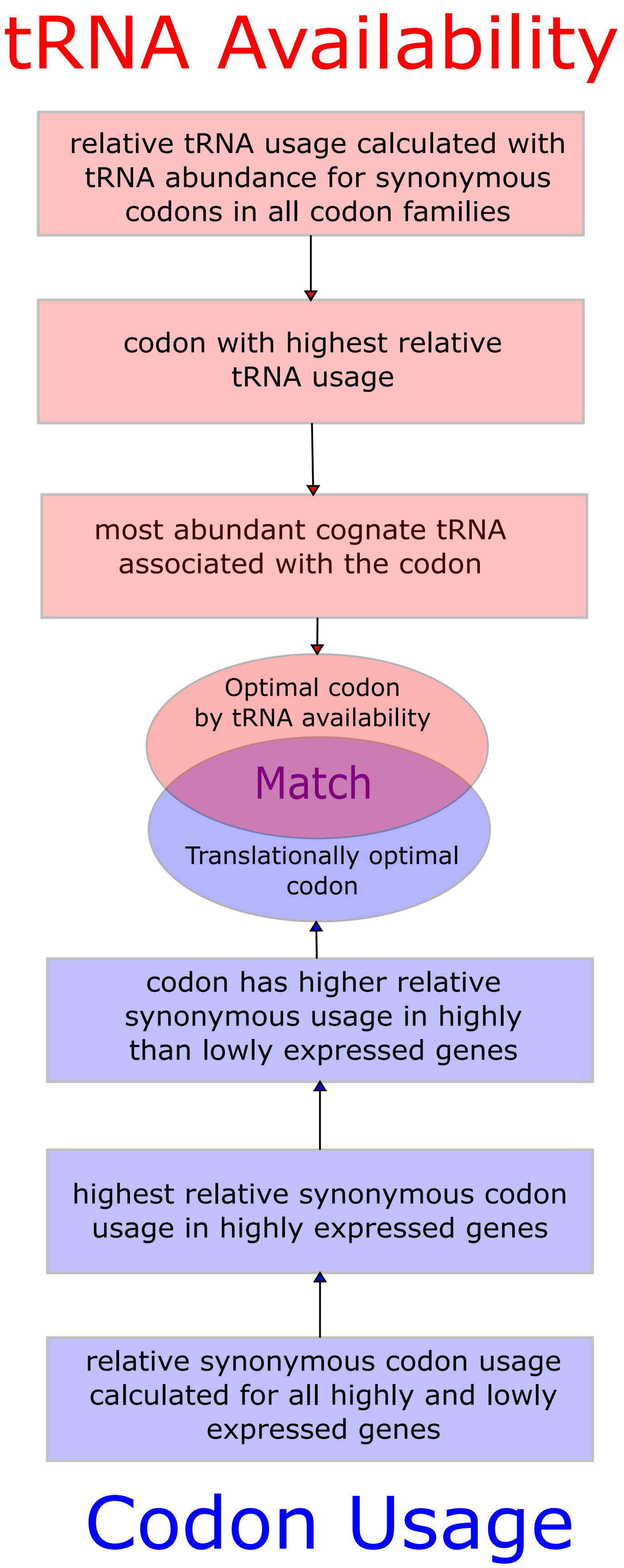

Supplementary Figure S1. Flowchart demonstrating the optimal codon designation through both tRNA availability and codon usage.

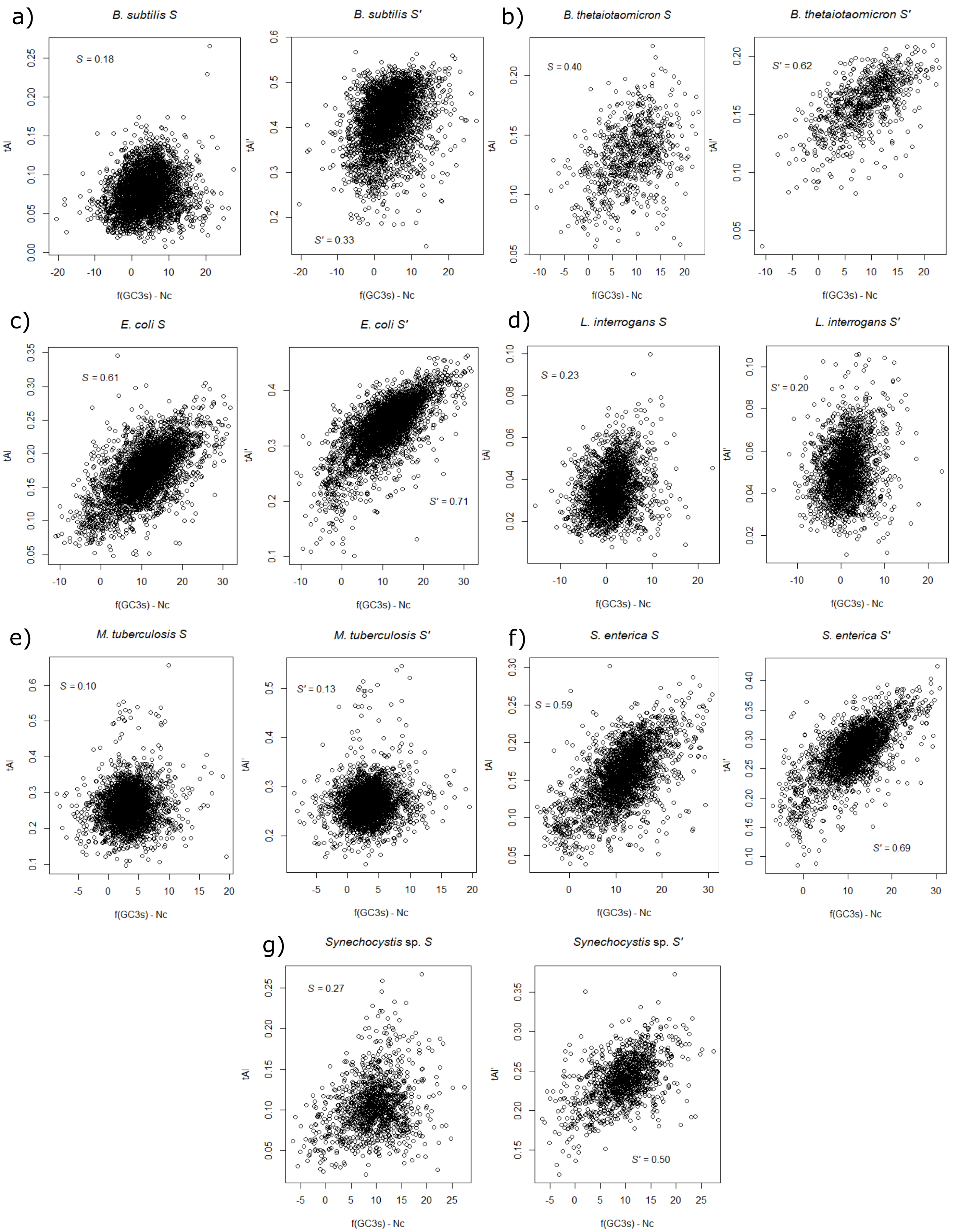

Supplementary Figure S2. The relationship between the corrected Nc and tAI/tAI' values for all seven species. The S or S' correlation coefficients are shown on each plot. Within each species, tAI and tAI' differ significantly from one another ($p < 0.0001$).

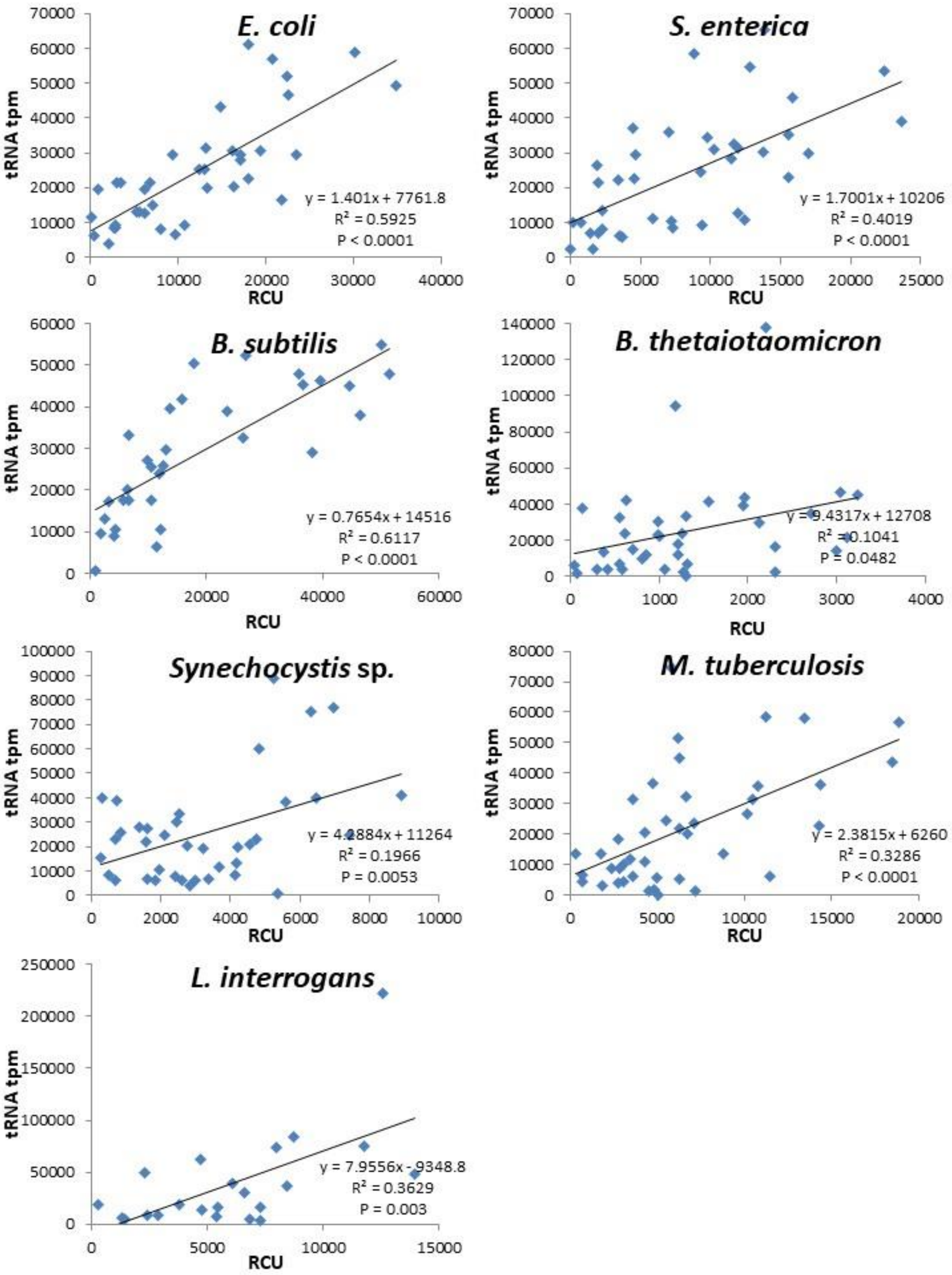

Supplementary Figure S3. Relationship between readable codon usage (RCU) and tRNA tpm in HEGs determined by protein per transcript, in three fast-growing species and four slow-growing species.

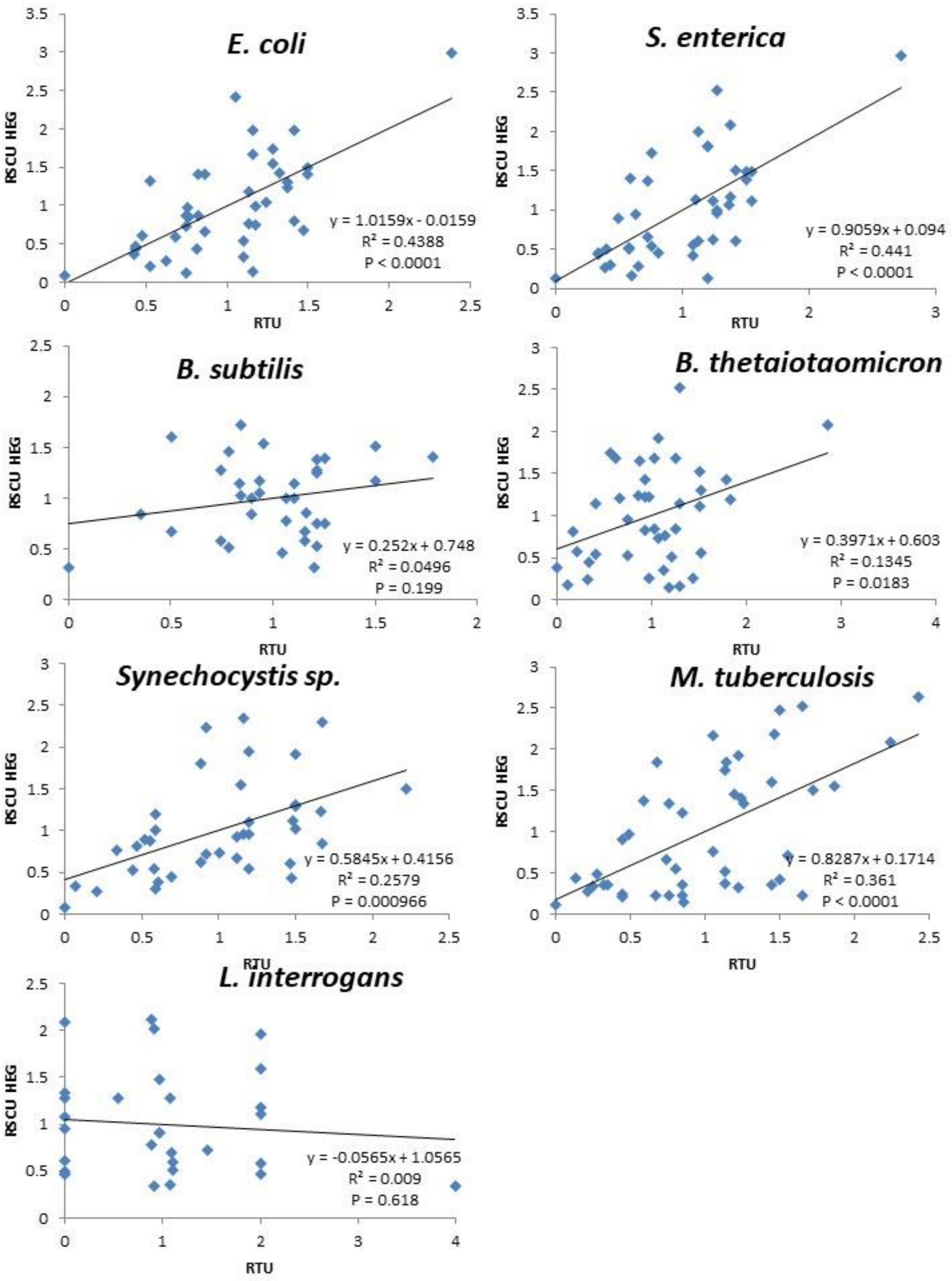

Supplementary Figure S4. Relationship between relative tRNA tpm usage (TPU) and RSCU in HEGs determined by protein per transcript, in three fast-growing species and four slow-growing species. Synonymous codons with RTU = 1 are due to lack difference in total readable (cognate and wobble pairing) tRNA tpm, they are omitted.

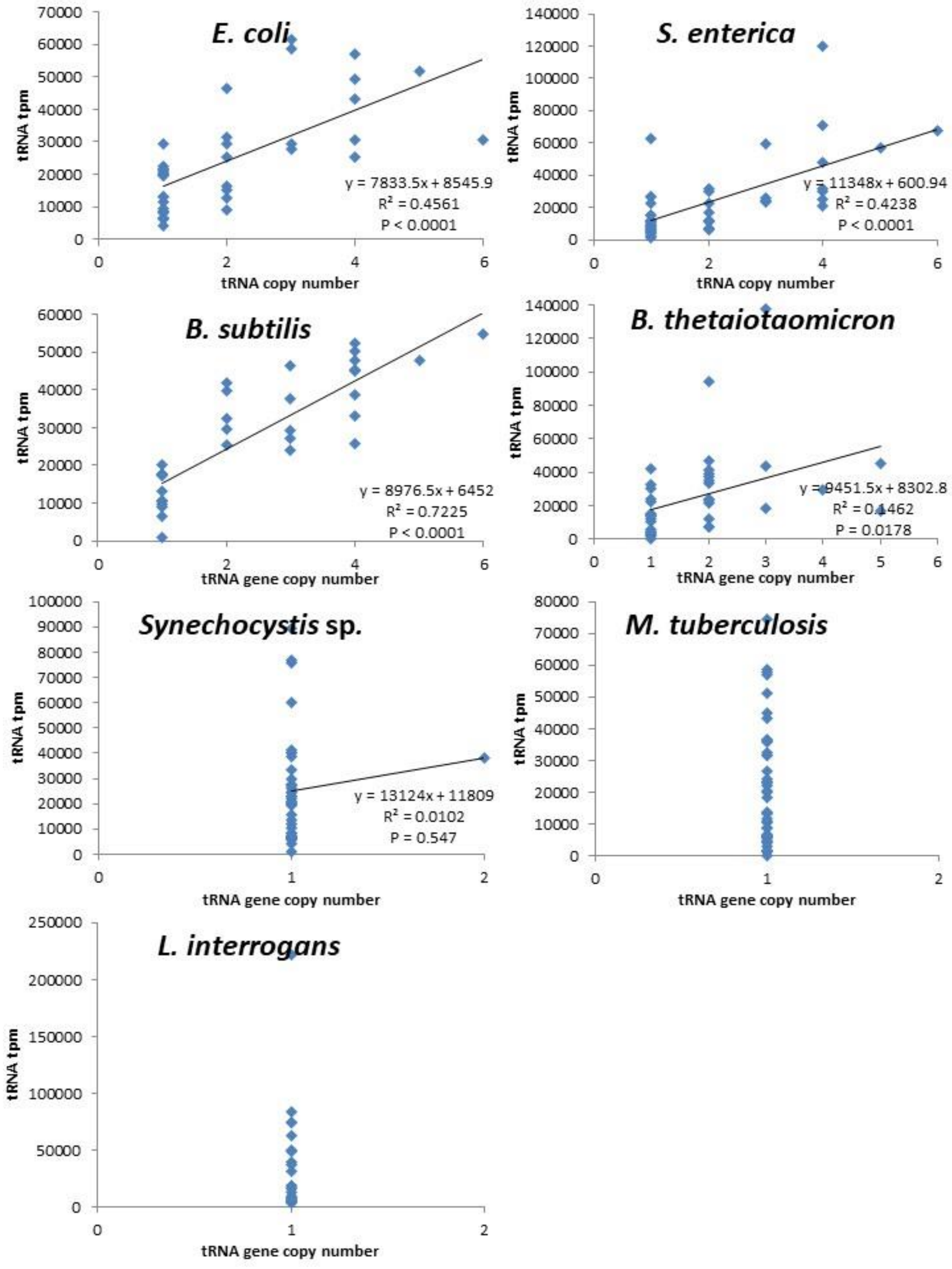

Supplementary Figure S5. Relationship between relative tRNA gene copy number and tRNA tpm values in three fast-growing species and four slow-growing species.

Supplementary Table S1. Number of genes determined to be translationally highly expressed (HEG) and lowly expressed (LEG) based on protein per transcript. The selected HEGs and LEGs are all genes found in the top and bottom 30% of genes by protein per transcript, respectively, for both datasets of each species (by SRX ID). The numbers of ribosomal protein genes contained in top and bottom 30% ppm/tpm gene sets are also shown for each dataset (by SRX ID).

		**Number of genes**		**Ribosomal protein genes**			**Matching genes between the both gene sets**	
**Species**	SRX ID	Top 30% ppm/tpm	Bottom 30% ppm/tpm	Number of genes	In top 30% ppm/tpm	In bottom 30% ppm/tpm	Number of HEGs	Number of LEGs
E. coli	SRX515174	1110	1110	57	50	0	976	962
	SRX669653	1119	1119		55	0		
B. subtilis*	SRX515181	1056	1056	57	42	0	1056	1056
	SRX2804667	N/A	N/A		23	0		
B. thetaiotaomicron	SRX020805	186	186	55	17	5	114	150
	SRX860738	230	230		24	1		
L. interrogans	SRX2448246	735	735	54	39	1	505	543
	SRX405952	672	672		31	1		
M. tuberculosis	SRX1372108	995	995	58	48	4	866	901
	SRX4374910	996	996		48	3		
S. enterica	SRX1638989	866	866	59	45	1	649	646
	SRX1258668	784	784		52	0		
Synechocystis sp.	SRX347145	400	400	55	43	1	306	308
	SRX4145044	360	360		42	1		

*- *B. subtilis* SRX2804667 was not considered due to Illumina MiSeq protocol removing large RNA transcripts (e.g. protein-coding gene transcripts).
